# ATM Influences the Efficiency of TCRβ Rearrangement, Subsequent TCRβ-Dependent T Cell Development, and Generation of the Pre-Selection TCRβ CDR3 Repertoire

**DOI:** 10.1371/journal.pone.0062188

**Published:** 2013-04-23

**Authors:** Karen S. Hathcock, Steven Bowen, Ferenc Livak, Richard J. Hodes

**Affiliations:** 1 Experimental Immunology Branch, National Cancer Institute, National Institutes of Health, Bethesda, Maryland, United States of America; 2 Department of Microbiology and Immunology, University of Maryland School of Medicine, Baltimore, Maryland, United States of America; Université Libre de Bruxelles, Belgium

## Abstract

Generation and resolution of DNA double-strand breaks is required to assemble antigen-specific receptors from the genes encoding V, D, and J gene segments during recombination. The present report investigates the requirement for ataxia telangiectasia-mutated (ATM) kinase, a component of DNA double-strand break repair, during TCRβ recombination and in subsequent TCRβ-dependent repertoire generation and thymocyte development. CD4^−^CD8^−^ double negative stage 2/3 thymocytes from ATM-deficient mice have both an increased frequency of cells with DNA break foci at TCRβ loci and reduced Vβ-DJβ rearrangement. Sequencing of TCRβ complementarity-determining region 3 demonstrates that ATM-deficient CD4^+^CD8^+^ double positive thymocytes and peripheral T cells have altered processing of coding ends for both in-frame and out-of-frame TCRβ rearrangements, providing the unique demonstration that ATM deficiency alters the expressed TCRβ repertoire by a selection-independent mechanism. ATMKO thymi exhibit a partial developmental block in DN cells as they negotiate the β-selection checkpoint to become double negative stage 4 and CD4^+^CD8^+^ thymocytes, resulting in reduced numbers of CD4^+^CD8^+^ cells. Importantly, expression of a rearranged TCRβ transgene substantially reverses this defect in CD4^+^CD8^+^ cells, directly linking a requirement for ATM during endogenous TCRβ rearrangement to subsequent TCRβ-dependent stages of development. These results demonstrate that ATM plays an important role in TCRβ rearrangement, generation of the TCRβ CDR3 repertoire, and efficient TCRβ-dependent T cell development**.**

## Introduction

Antigen-specific receptors expressed by T and B cells are heterodimers encoded by genes that must first be assembled from variable (V), diversity (D), and (J) joining gene segments by recombination. V(D)J recombination is initiated when RAG-1/2 proteins generate DNA double-strand breaks (DSB) between recombination signal sequences and adjacent V, D, or J gene segments, producing blunt-ended signal ends (SE) and coding ends (CE) with terminal hairpin loops. Repair of the CE by non-homologous end joining (NHEJ) re-establishes the intact, but rearranged chromosome necessary for expression of antigen-specific receptor proteins [1], [2]. Due to the imprecise nature of NHEJ, the junctional region of the recombined V, D, and J segments exhibits large sequence variations which encode the highly diverse, antigen-binding, complementarity-determining region 3 (CDR) of antigen receptor chains [3], [4].

Rearrangement of the TCR β, γ and δ chains occurs early in thymic T cell development during the RAG1/2-positive CD4^−^CD8^−^ double negative (DN) 3 stage [5]–[7]. Only DN3 cells that successfully rearrange and express a TCRβ chain can form a pre-TCR and negotiate the β-selection checkpoint to become DN4 and CD4^+^CD8^+^ (DP) thymocytes [8]–[10]. Re-expression of RAG1/2 in DP cells allows TCRα chain rearrangement [11] and subsequent surface expression of an antigen-specific TCRαβ heterodimer on DP cells. TCRαβ^+^ DP cells that survive selection generate mature TCRαβ^+^ CD4^+^ or CD8^+^ single positive (SP) T cells that recognize a large universe of foreign, but not self-antigens [12]. Therefore, factors that influence V(D)J rearrangement play critical roles in T cell development and in establishing the expressed antigen-specific T cell repertoire.

The protein product of the ataxia telangiectasia-mutated (ATM) gene is a kinase critical for sensing and responding to DNA DSB in a variety of circumstances, including V(D)J recombination [13]–[19]. Phenotypes of both AT patients and ATMKO mice as well as other experimental approaches have demonstrated the importance of ATM in facilitating V(D)J recombination. ATM deficiency in humans and mice results in lymphopenia, increased genomic instability, and increased incidence of T cell malignancies that have translocations involving TCR loci [20]–[22]. Our laboratory and others demonstrated that when ATM is deficient, TCRβ rearrangement is impaired in DP cells and is associated with an accumulation of unrepaired CE and reduced numbers of SP cells [23], [24]. ATM deficiency is also associated with unresolved CE during TCRβ, γ and δ rearrangement and impaired TCRβ rearrangement [25], [26].

This present report investigates requirements for ATM during murine TCRβ rearrangement and in subsequent stages of TCRβ-dependent repertoire generation and development. As compared to ATMWT DN2/3 cells, ATMKO cells have an increased frequency of cells with 53 BP1 DNA damage foci at the TCRβ locus and reduced V(D)Jβ recombination. In addition, ATM deficiency alters the expressed TCRβ repertoire by a selection-independent effect on CDR3 sequences that results from modified processing of CE. Defective TCRβ rearrangement in ATMKO DN2/3 cells correlates with a partial developmental block in the ability of DN3 cells to negotiate the β-selection checkpoint to become DN4 and DP cells, resulting in reduced numbers of DP cells. Importantly, expression of a TCRβ transgene (TG) in the ATMKO substantially reverses this defect in DP cell numbers, directly linking these TCRβ-dependent developmental defects to the requirement for ATM during endogenous TCRβ rearrangement.

## Results

### Resolution of DNA DSB Foci and Rearrangement at TCRβ are Defective in ATMKO DN2/3 Cells

**Figure 1:**
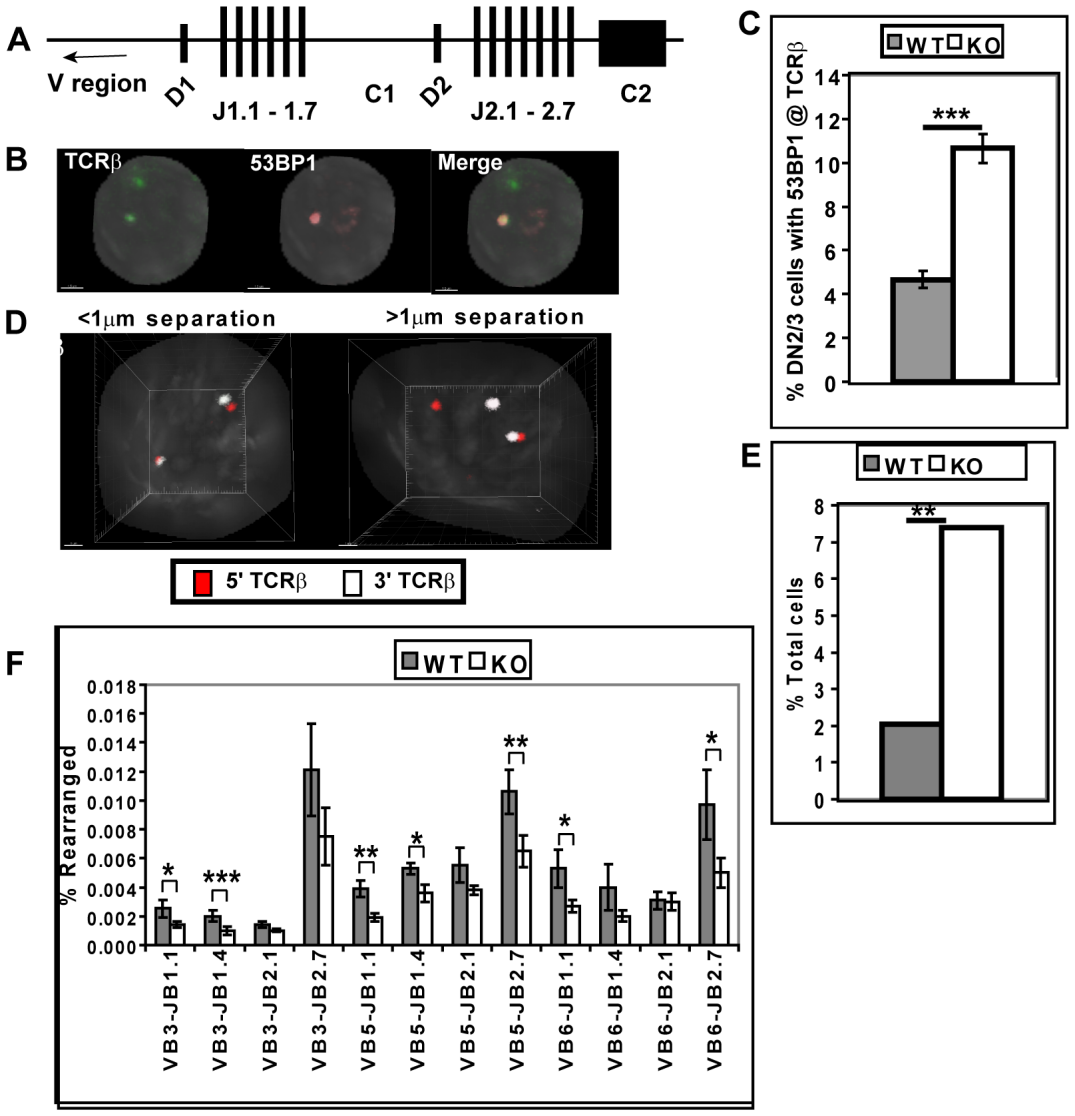
Rearrangement of TCRβ is impaired in ATMKO DN2/3 cells. (A) Genomic organization of the murine TCRβ locus. (B) A representative immuno-FISH image of an ATMKO DN2/3 cell showing a 53 BP1 focus (red) at one of two TCRβ loci (green). White bar denotes 1.5 micron. (C) Graph shows mean frequency (±SEM) of ATMWT (WT) and ATMKO (KO) DN2/3 cells expressing 53 BP1 foci at the TCRβ locus. 200 cells of each genotype were analyzed in two experiments. (D) Representative 3D-FISH images of two ATMKO DN2/3 cells hybridized with 5′TCRβ (red) and 3′TCRβ probes (white). In left image probes are separated by less than 1 µm at both TCRβ loci; whereas, in right image probes are separated by more than 1 µm at one of two TCRβ loci. (E) Summary of the frequency of ATMWT and KO DN2/3 cells in which the 5′ and 3′TCRβ probes are separated by more than 1 µm one of two TCRβ loci. More than 300 cells of each genotype were collected in two experiments. (F) Vβ-DJβ rearrangement in genomic DNA from ATMWT and KO DN2/3 cells was quantified using real-time PCR and normalized to invariant Cα. This plot is mean Vβ-DJβ rearrangements±SEM of seven ATMWT and KO data sets analyzed in five real-time PCR experiments. P values: Student’s unpaired (C) and paired (F) 1-tailed t-test and Fisher’ exact test (E). *p<0.05; **<0.01, ***p<0.001.

We first assessed ATM function in ATMWT and KO DN2/3 thymocytes using FISH and immunofluorescence staining (immuno-FISH) to visualize and quantify DSB foci at the TCRβ locus. Specifically, DSB at the TCRβ locus were identified by hybridization with a probe specific for the 5′ end of the TCRβ locus and staining for the DNA damage response molecule 53 BP1 [27], [28]. In DN2/3 cells 53 BP1 foci co-localized with the TCRβ locus in 4.7% of ATMWT cells and with a substantially increased 10.7% of ATMKO cells (Fig. 1 B–C). In both ATMWT and KO DN2/3 cells, >90% of 53 BP1 foci were localized to the TCR β, γ and δ loci that rearrange in DN2/3 cells. In ATMWT and KO DN2/3 cells that are also RAG1-deficient there were no detectable 53 BP1 foci co-localizing with TCRβ in 0/370 ATMWT or 0/396 ATMKO cells examined, reinforcing the conclusion that even when ATM is deficient, 53 BP1 foci are primarily restricted to rearranging TCR loci. Since persistent DNA damage foci in irradiated ATMKO cells have been shown to correlate with persistence of DNA damage [29], the increase in 53 BP1 foci at TCRβ loci reported here may similarly reflect a requirement for ATM for efficient resolution of RAG-induced DSB.

To further assess the integrity of the TCRβ locus in ATMWT and KO DN2/3 thymocytes, we used 3-dimensional FISH (3D-FISH) with probes specific for the 5′ and 3′ ends of the TCRβ locus. We measured the 3-dimensional distances between the 5′ and 3′ ends of the TCRβ locus and used a 1 µm distance to distinguish intact loci (<1 µm) from loci in which the 5′ and 3′ ends are physically separated (>1 µm) [30]. Physical separation of the TCRβ locus, an indicator of genomic instability at the locus, can result from mobile broken DNA ends, chromosomal translocation, or de-contracted chromosomes [19], [31], [32]. Only 4/196 ATMWT DN2/3 cells (2.0%) contained at least one TCRβ allele where the distance between these TCRβ probes was greater than 1 µm. In contrast, 7.4% (14/189 cells) of ATMKO DN2/3 cells contained at least one TCRβ allele where the distance between probes was greater than 1 µm (Figure 1D–E). Therefore, ATM deficiency results in TCRβ instability as defined by physical separation of 5′ and 3′ ends of the locus.

To directly assess the requirements for ATM during TCRβ rearrangement we quantified Vβ-DJβ rearrangement in ATMWT and KO DN2/3 cells by real-time PCR (Figure 1F). As compared to ATMWT cells, Vβ-DJβ rearrangements in ATMKO cells were reduced in all 12 Vβ-DJβ combinations analyzed, and these differences reached statistical significance in 7 of the 12 Vβ-DJβ combinations. Thus, ATM deficiency also results in reduced TCRβ rearrangement in DN2/3 cells.

### ATM Deficiency Alters Junctional TCRβ CDR3 Sequences in Pre- and Post-selection T cells

**Figure 2:**
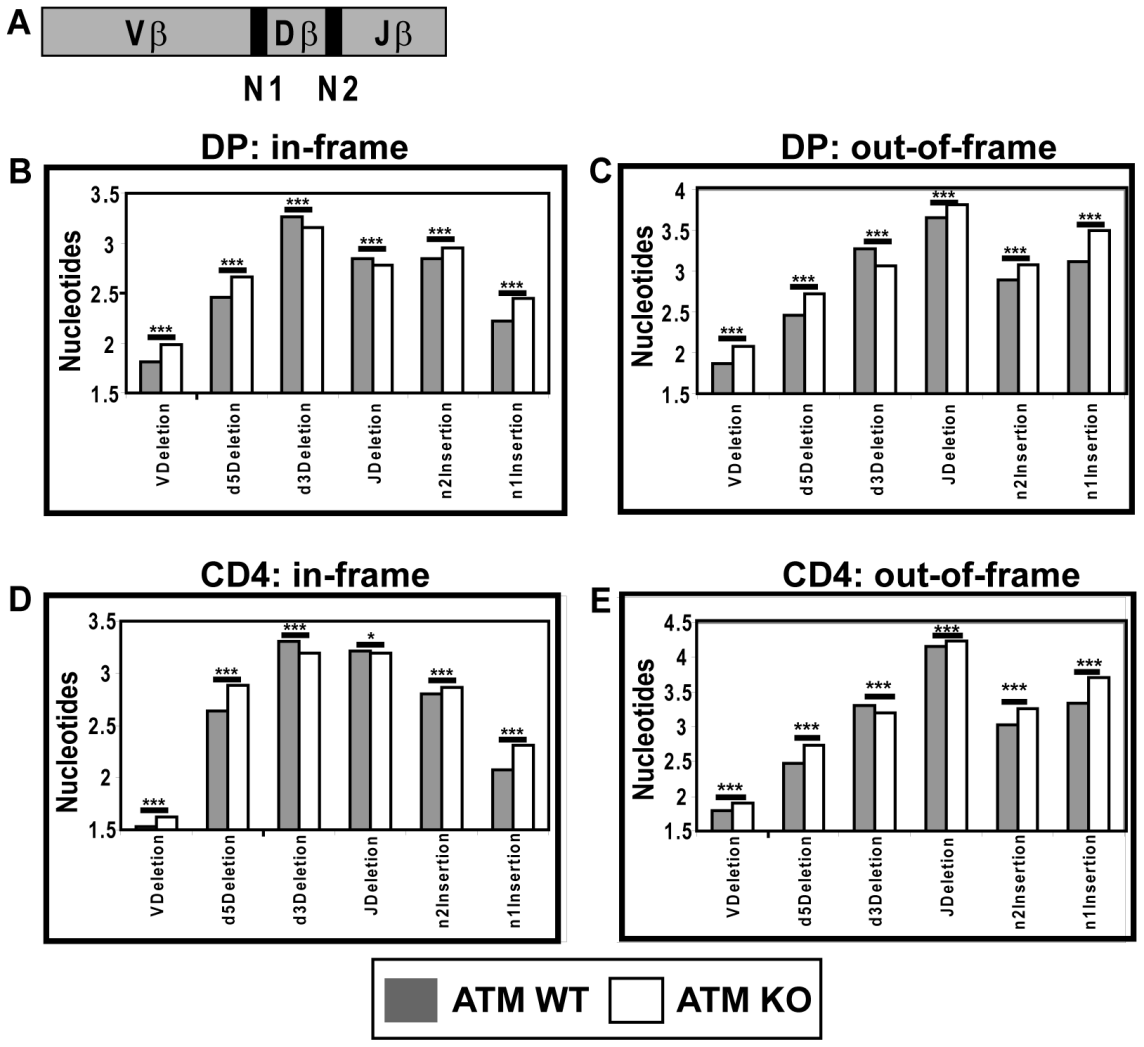
ATM deficiency alters in-frame and out-of-frame junctional sequences of TCRβ CDR3 in pre- and post-selection T cells. (A) Diagram of TCRβ CDR3 junctions. Nucleotide deletions and additions were sequenced from in-frame and out-of-frame TCRβ CDR3 regions isolated from ATMWT (grey bars) or KO (white bars) cells that were either TCRα KO DP thymocytes (B and C) or naïve CD4^+^ T cells (D and E). TCRαKO DP and naïve CD4^+^ T cells were sorted from two mice of each ATM genotype. Each data point represents >55,000 sequences. P values were calculated using Student’s unpaired 2-tailed t-test. *p<0.05; **p<0.01; ***p<0.001.

Antigen specificity is in large part determined by the CDR3 region that spans the V-D-J junction of TCRβ chains [3], [4]. Having shown that ATM is required for efficient resolution of TCRβ DSB and subsequent TCRβ rearrangement, we asked whether ATM also affects the junctional sequences of TCRβ CDR3 regions. We first used high throughput sequencing to identify and characterize in-frame CDR3 sequences from TCRβ-deficient ATMWT or KO DP cells. TCRαKO DP cells express TCRβ but no TCRα and thus do not express TCRαβ heterodimers and are not subject to selection based on TCRαβ specificity [33]. CDR3 sequences from TCRαKO ATMWT or TCRαKO ATMKO DP cells did not differ in overall length. However, the number of nucleotides deleted from the coding flanks of 3′Vβ, 5′Dβ, 3′Dβ and 5′Jβ genes as well as the number of template independent N nucleotides present at the Vβ-Dβ (N1) and Dβ-Jβ (N2) junctions were significantly and consistently different between ATMWT and KO T cells (Figure 2 B–C). Specifically, in-frame TCRβ CDR3 junctions from TCRαKO ATMKO DP cells had consistently more nucleotides deleted from the 3′Vβ and 5′Dβ coding flanks and fewer nucleotides deleted from the 3′ Dβ and 5′Jβ regions. In addition, the average number of N nucleotides added at both Vβ-Dβ (n1 insertions) and Dβ-Jβ (n2 insertions) was also increased in the ATMKO samples. We next asked if these differences in TCRβ CDR3 modifications that were detected in unselected ATMWT and KO DP cells were also maintained in naïve peripheral CD4^+^ cells that express a selected TCRαβ repertoire (Figure 2 D–E). The differences in TCRβ CDR3 sequences detected in unselected DP cells were maintained with striking consistency in post-selection CD4^+^ T cells. In addition, with the exception of the 5′Jβ region, out-of-frame sequences obtained from ATMWT and KO DP and CD4^+^ T cells identified modifications of TCRβ CDR3 regions that were identical to the modifications detected for in-frame sequences. Specifically, in-frame 5′Jβ sequences from ATMKO cells had consistently fewer nucleotides deleted than did WT sequences while out-of-frame 5′Jβ regions from ATMKO cells had more nucleotides deleted than WT did. These results provide the unique demonstration that the expressed TCR repertoire, specifically the TCRβ-dependent CDR3 repertoire, is influenced by ATM and that this influence occurs through a selection-independent mechanism.

### ATM-deficiency Impairs Development through the β-selection Checkpoint

**Figure 3:**
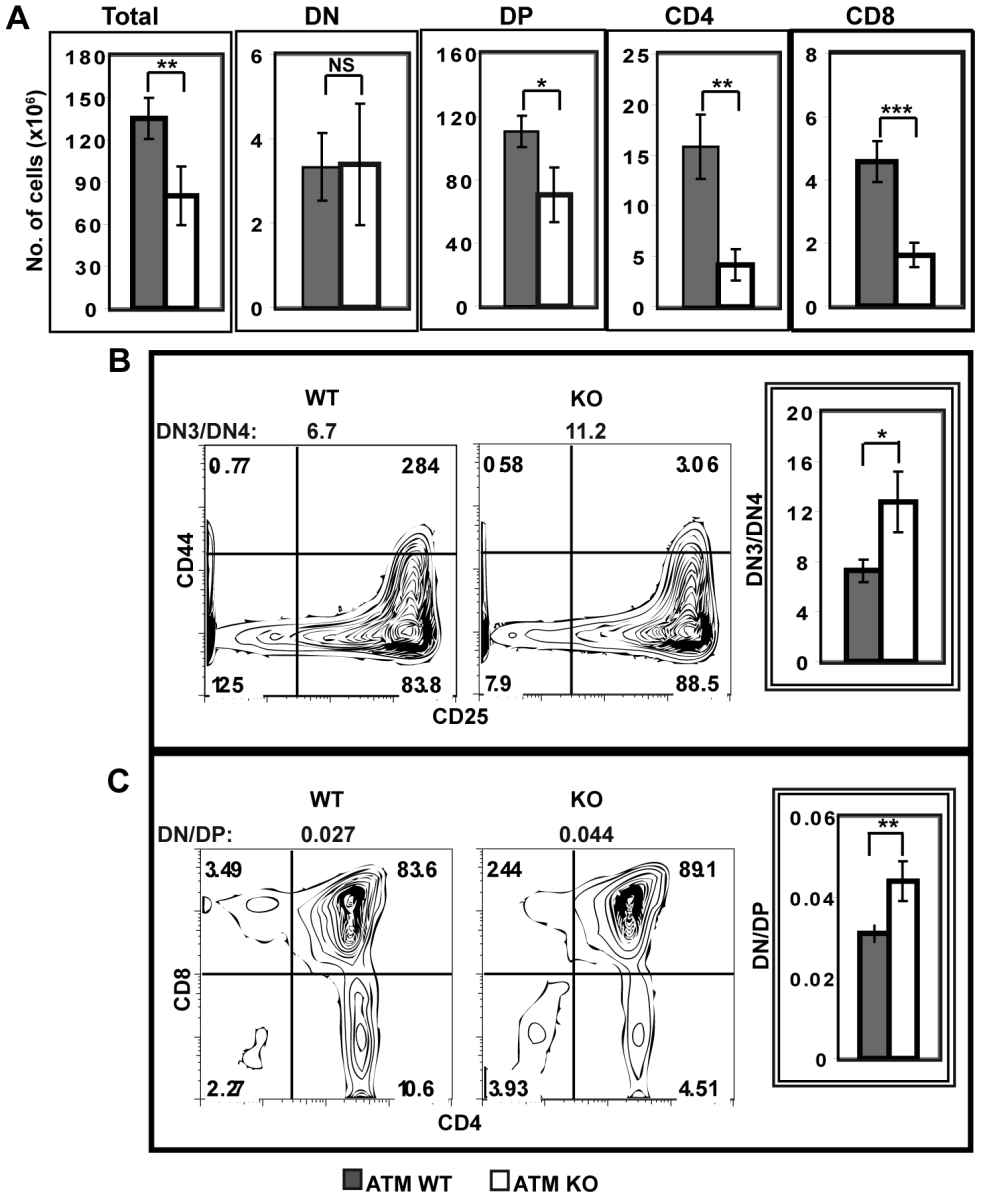
ATM deficiency alters thymic development. (A) Thymocytes from four pairs of ATMWT and KO mice were analyzed by flow cytometry to enumerate cells in each CD4/CD8 subpopulation. (B) The DN3/DN4 ratio is elevated in ATMKO thymi. Representative CD44 and CD25 staining profiles of lineage negative ATMWT and KO DN cells and the calculated DN3/DN4 ratios are shown. Quantification of DN3/DN4 ratios measured in ATMWT and KO thymi (ATMWT vs KO p<0.025). (C) The DN/DP ratio is elevated in ATMKO thymi. Images show CD4/CD8 staining profiles and corresponding DN/DP ratios from representative ATMWT and KO thymi. Quantification of DN/DP ratios measured in ATMWT and KO thymi (ATMWT vs KO p<0.003). Data are mean (± SEM) of six separate experiments analyzing nine pairs of ATMWT and KO thymi. P values were calculated using Student’s paired 1-tailed t test.

As compared to ATMWT thymi, ATMKO thymi have reduced cellularity and reduced numbers of DP and SP thymocytes, but no change in the number of DN cells (Figure 3A). We asked if the phenotype of ATMKO thymi is solely the consequence of the previously demonstrated defect in TCRα rearrangement [23], [24] or if the defects described here in TCRβ rearrangement also contribute to this phenotype. Since TCRβ rearrangement occurs in DN3 (CD25^+^CD44^−^) cells, and only DN3 cells that successfully rearrange TCRβ and express a pre-TCR can differentiate to DN4 (CD25^−^CD44^−^) and DP cells, we analyzed the ratios of DN3/DN4 and DN/DP cells in ATMWT and KO thymi. As compared to ATMWT thymi, both the DN3/DN4 ratio (6.7 in WT vs. 11.2 in KO, p<0.03) (Figure 3B), and the DN/DP ratio (0.027 in WT vs. 0.044 in KO, p<0.003) (Figure 3C) in ATMKO thymi are reproducibly elevated, indicating a decreased efficiency of ATMKO cells to transition through the β-selection checkpoint. These results demonstrate that ATM is required for optimal thymic development from DN3 cells through the β-selection checkpoint to DN4 and DP cells.

### ATM Deficiency Alters the Dynamics of Cell Survival and Cell Division in DN Cells

**Figure 4:**
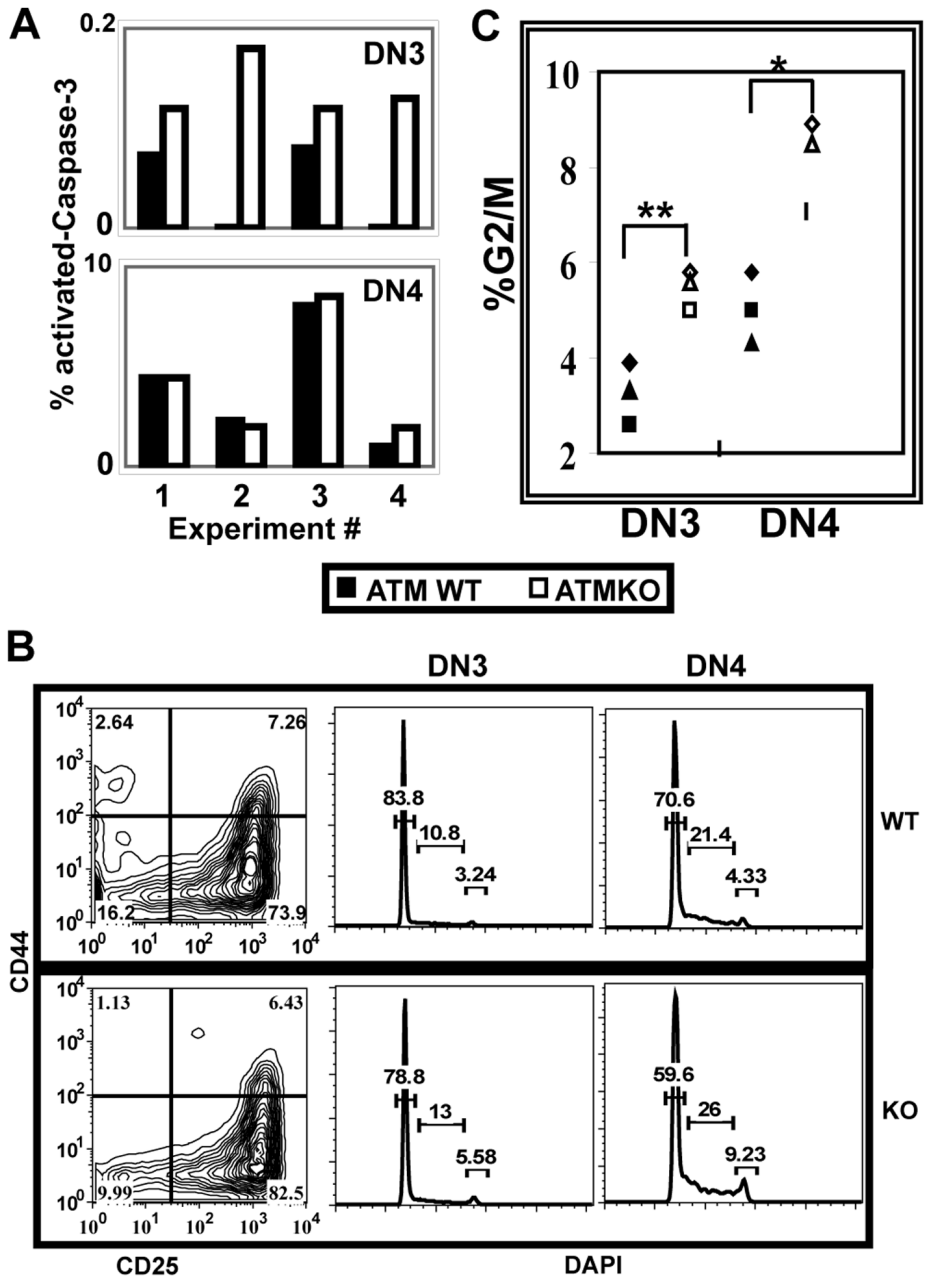
ATM deficiency alters cell survival and proliferation in DN cells. Freshly explanted thymocytes were stained for surface molecules, fixed, and permeabilized prior to intracellular staining. **(**A) As compared to ATMWT, ATMKO DN3 but not DN4 cells have an increased frequency of cleaved-Caspase-3^+^ cells (ATMWT vs KO DN3 p<0.03). Graphs show frequencies of ATMWT (grey bar) and KO (white bar) lineage negative DN3 and DN4 cells that express cleaved-Caspase-3. Data from four independent experiments analyzing ATMWT and KO pairs is shown. (B) Panels show representative lineage negative CD25 CD44 DN staining profiles for ATMWT (top panel) and KO (lower panel) thymi and DAPI staining profiles gated on DN3 and DN4 cells. (C) Frequency of DN3 and DN4 cells from individual ATMWT (black symbols) and KO (open symbols) mice that are in G2/M of the cell cycle. ATM deficiency results in increased cycling cells in both DN3 and DN4 stages (p<0.002 and 0.02, respectively). P values were calculated using Student’s paired 1-tailed t test from data collected from three-four ATMWT and KO pairs analyzed in three independent experiments.

Since successful TCRβ rearrangement results in extensive proliferation and prolonged survival of DN thymocytes, we compared cell survival and cell division in ATMWT and KO DN cells. We first analyzed expression of intracellular cleaved-Caspase-3 as an indicator of apoptotic cell death in DN3 and DN4 cells [34], [35]. The frequency of activated-Caspase-3^+^ cells was quite low in DN cells regardless of ATM genotype; however, as compared to ATMWT, the frequency of activated-Caspase-3^+^ cells was consistently and significantly elevated in ATMKO DN3 cells (p<0.03), where TCRβ is rearranging. In contrast there was no significant difference in the frequency of activated-Caspase-3^+^ cells between ATMWT and KO DN4 cells which have largely completed rearrangement (Figure 4A).

We next used DAPI staining to assess the dynamics of cycling DN3 and DN4 cells in ATMWT and KO thymi (Figure 4B). During normal DN development, the frequency of cells in G2/M of the cell cycle increases between DN3 and DN4 stages as cells rearrange TCRβ and express a pre-TCR, allowing the successful negotiation of the β-selection checkpoint [36]. Thus in both ATMWT and KO thymi, the frequency of cells in G2/M increases as cells transition from DN3 to DN4. However, as compared to ATMWT DN cells, both DN3 and DN4 cells from the ATM KO thymi have significantly increased frequencies of cells in G2/M (Figure 4C). Taken together these results demonstrate that ATM deficiency alters the cellular kinetics of the DN compartment, increasing both the frequency of apoptotic DN3 cells and the frequencies of cycling DN3 and DN4 cells. Since the total number of DN cells does not differ between ATMWT and KO thymi (Figure 3A), the net effect of the increased cell death in DN2/3 cells and the increased cell cycling in DN3 and DN4 cells is maintenance of ATMKO DN cell numbers, but with an increased DN3/DN4 ratio reflecting the defect in transition through the β-selection checkpoint.

### The Developmental Defect in ATM-deficient T cells is Cell-intrinsic

**Figure 5:**
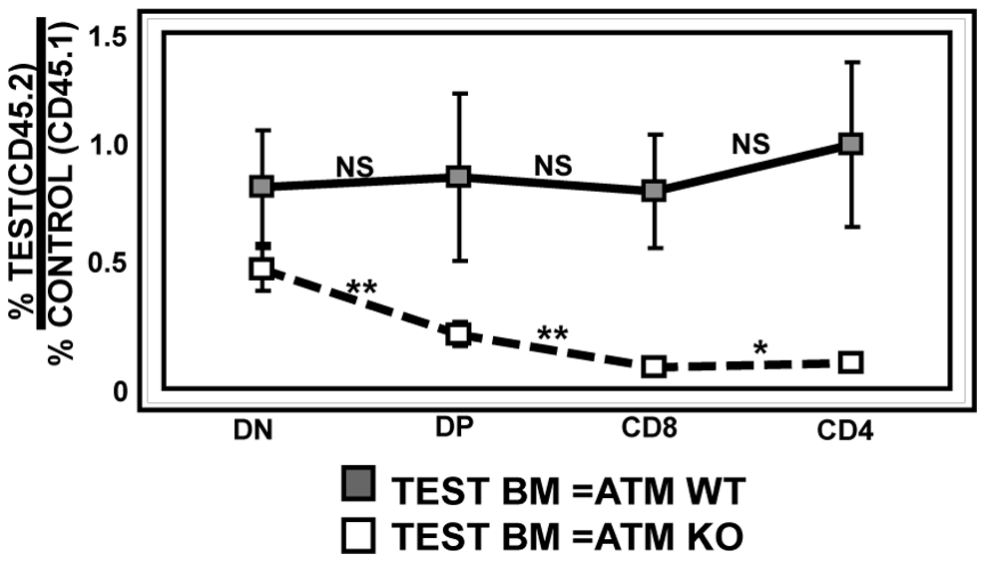
Competitive chimeras demonstrate that the ATMKO defect in DN to DP development is T cell-intrinsic. This graph shows the ratio of thymocytes derived from test (ATMWT or KO CD45.2^+^) and control (ATMWT CD45.1^+^) bone marrow for each thymic subset. When both test (CD45.2^+^) and control (CD45.1^+^) bone marrows are ATMWT (solid black line), the ratio of test/control cells is not significantly changed during thymic development. In contrast, when test bone marrow is ATMKO (CD45.2^+^) (dashed black line) the ratio of test/control (ATMKO/ATMWT) cells decreases as thymocytes develop from DN to DP cells (p<0.007) and from DP to CD8 (p<0.005) or CD4 (p<0.02) SP cells. Data from two independent sets of chimeras consisting of 16–20 mice in each group were combined for this analysis. P values were calculated using Student’s unpaired 1-tailed t-test.

We next used competitive bone marrow chimeras to directly compare the efficiency of ATMWT and KO bone marrow cells to reconstitute the T cell lineage. In chimeras reconstituted with equal numbers of CD45.1^+^ and CD45.2^+^ ATMWT donor cells, the ratio of CD45.2^+^/CD45.1^+^ cells (ATMWT/ATMWT) detected in each thymic subset was relatively unchanged as thymocytes developed from DN to DP and from DP to SP cells, indicating that the differentiation capacities of the two ATMWT (CD45.1^+^ and CD45.2^+^) donor bone marrow populations were equivalent throughout thymic development. In contrast, in chimeras reconstituted with a mixture of ATMWT (CD45.1^+^) and ATMKO (CD45.2^+^) bone marrow cells, the ratio of CD45.2^+^/CD45.1^+^ (ATMKO/ATMWT) cells decreased as cells differentiated from DP to SP cells, demonstrating the competitive disadvantage of ATMKO cells to develop from DP to SP cells. In addition, the (CD45.2^+^/CD45.1^+^) (ATMKO/ATMWT) ratio also decreased as thymocytes developed from DN to DP cells, demonstrating that ATMKO thymocytes also have an additional competitive disadvantage as they develop from DN to DP (Figure 5). This result confirms the existence of the DN to DP developmental block detected in ATMKO thymocytes and further demonstrates that this block is T cell-intrinsic.

### Expression of a Rearranged TCRβ TG Overcomes the Block at the β-selection Checkpoint in ATMKO Thymocytes

**Figure 6:**
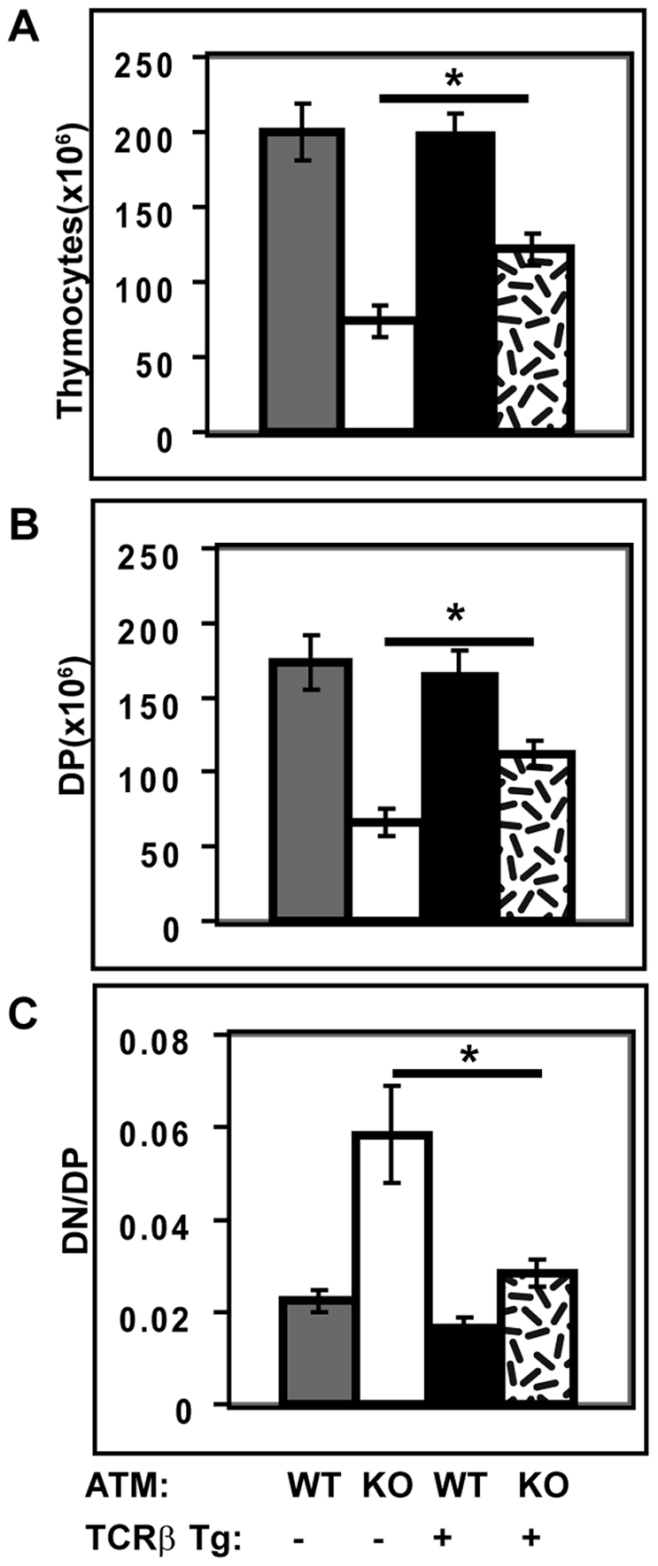
Introduction of a rearranged TCRβ TG significantly improves the ATMKO defect in DN to DP development. Effects of TCRβ TG expression on ATMWT (WT) and ATMKO (KO) thymic cellularity (A), numbers of DP cells (B), and DN/DP ratios (C). Results in panels A–C are mean (± SEM) for three ATMWT and KO pairs analyzed in three independent experiments. For the parameters that were tested, ATMWT thymi with and without the TCRβ TG did not differ significantly. In contrast, ATMKO thymi with and without the TCRβ TG did differ significantly (p<0.05). P values were calculated using Student’s paired 1-tailed t test.

Impaired T cell development through the β-selection checkpoint is consistent with a requirement for ATM during V(D)Jβ rearrangement in DN3 cells, but it could also reflect a requirement for ATM that is independent of V(D)Jβ rearrangement. In order to distinguish between these possibilities we introduced a rearranged TCRβ TG into ATMWT or KO mice. The TCRβ TG pairs with endogenous pre-Tα to form a pre-TCR and allows development through the β-selection checkpoint in the absence of a requirement for endogenous TCRβ rearrangement [23], [37], [38]. All previous experiments that tested the requirements for ATM during TCR rearrangement used TCRαβ TG that express both a rearranged TCRα and β TG and consequently could not distinguish between TCRα- and TCRβ-dependent effects on development [23], [37]. Introduction of only a TCRβ TG specifically tests the requirement for endogenous TCRβ rearrangement. Expression of the TCRβ TG in ATMWT thymi did not significantly change thymic cellularity, numbers of DP cells, or the DN/DP ratio. In marked contrast, expression of the TCRβ TG in ATMKO thymi significantly increased thymic cellularity and numbers of DP cells. In addition, the DN/DP ratio was reduced in the TCRβ TG^+^ ATMKO thymi, reflecting improved development through the β-selection checkpoint (Figure 6A–C). These results demonstrate that in ATM-deficient thymi the developmental block at the β-selection checkpoint is significantly reversed when development is no longer dependent on endogenous TCRβ rearrangement and that the requirement for ATM at the β-selection checkpoint is substantially due to the requirement for ATM in V(D)Jβ rearrangement.

## Discussion

The studies presented here demonstrate requirements for ATM in TCRβ rearrangement, TCRβ repertoire generation, and TCRβ-dependent T cell development. Specifically, we show that ATM deficiency compromises TCRβ rearrangement and alters the expressed TCRβ CDR3 repertoire. Consistent with the observed defects in TCRβ rearrangement, ATM deficiency also inhibits TCRβ-dependent thymic differentiation through the β-selection checkpoint from DN3 to DN4 and DP cells. Importantly these early developmental defects are substantially reversed by expression of a rearranged TCRβ TG, directly linking the requirement for ATM during endogenous TCRβ rearrangement to defects in development at the β-selection checkpoint.

AT patients and ATMKO mice have reduced numbers of thymic and mature TCRαβ^+^ T cells. Previous studies demonstrated that ATM promotes efficient TCRα rearrangement in DP thymocytes and provided an explanation for reduced numbers of mature TCRαβ^+^ T cells associated with this deficiency [23], [24]. The present report identifies an additional requirement for ATM during TCRβ rearrangement that may also contribute to the reduction in mature T numbers. Since expression of only a TCRβ TG in the ATMKO significantly improves both the numbers of DP cells and the DN to DP transition, these results directly demonstrate that these defects in thymic development are in fact attributable to the requirement for ATM during TCRβ rearrangement. Thus, the findings presented here are consistent with previous reports demonstrating that ATM deficiency impairs resolution of DSB breaks at TCRβ [25] and development from DN3 to DN4 cells [26], and extends the interpretation of these associations by demonstrating that the developmental defect observed in early thymocyte development is in fact attributable to the requirement for TCRβ rearrangement. The fact that the TCRβ TG fails to completely reverse the ATM-deficient thymic phenotype is also of interest and is consistent with additional roles for ATM, including requirements for ATM during TCRα rearrangement or D-Jβ rearrangement that, in contrast to V(D)Jβ rearrangement, can occur in DP cells and is not suppressed by a TCRβ TG [39]. ATM-dependent effects on development that are independent of V(D)J rearrangement may also contribute to the thymic phenotype.

The function(s) of pre-TCR in the DN3 to DN4 transition is a subject of debate. Most models propose that the pre-TCR induces both proliferation and differentiation signals in DN3 thymocytes that are subsequently required for survival and negotiation of the β-selection checkpoint [8]–[10], [40]. However, an alternative model of pre-TCR function has been proposed that suggests the pre-TCR predominantly enhances survival, and is less important for proliferation, of DN3 thymocytes [41]. Our findings are consistent with this latter model indicating that pre-TCR may be more important in supporting survival rather than proliferation of developing DN cells. As compared to ATMWT DN cells ATMKO DN3 cells, but not post-β-selection DN4 cells, exhibited increased apoptosis; however, proliferation was not compromised in either ATMKO DN3 or DN4 cells. In fact in the ATMKO there are more DN3 and DN4 cells in cycle, despite ATM-dependent reductions in pre-TCR expression. This increase in cycling cells that is detected in the ATMKO is consistent with homeostatic compensation to maintain the DN3 and DN4 compartments [42] in the face of increased DN3 apoptosis and deficient DN3–DN4 transition. The net result of these changes in apoptosis and cycling cells is that the total number of DN cells is maintained in the ATM thymus. These findings do not exclude the possibility that ATM also enhances survival of DN3 thymocytes directly, by a pre-TCR-independent mechanism, possibly related to the proposed role of ATM in enhancing differentiation of lymphocyte precursors undergoing programmed gene rearrangement [16].

The findings reported here also uniquely demonstrate that ATM influences the TCRβ-dependent repertoire, as reflected by TCRβ CDR3 sequences. Spanning the V-D-J junction of TCRβ chains, the CDR3 region plays a critical role in determining antigen specificity [3], [4]. Sequencing the TCRβ CDR3 from ATMWT and KO T cells revealed consistently altered patterns of nucleotide additions and deletions in the absence of ATM. These differences in junctional modifications between ATMWT and KO were detected in both pre-selection TCRαKO DP cells and in post-selection naïve CD4^+^ T cells. In addition, differences in junctional modifications identified in ATMWT and KO in-frame sequences were identical to the modifications detected for out-of-frame sequences for all but the 5′Jβ region. Thus, ATM deficiency alters the TCRβ CDR3 repertoire by a selection-independent mechanism operating at the level of junctional diversification during VDJβ recombination. Our results suggest that ATM promotes efficient resolution of CE during TCRβ rearrangement and that when ATM is absent, CE are susceptible to altered processing by nucleotide addition and excision.

ATM is a multifunctional protein that coordinates a variety of responses to DNA DSB that are essential for maintaining genomic stability and preventing transformation [17], [18]. This ATM function also promotes V(D)J recombination in developing T and B cells, where ATM helps stabilize broken DNA ends and facilitates end joining [16], [25], [43]. Our observation that ATM deficiency alters the junctional diversity of the TCRβ repertoire raises a previously unrecognized possibility that ATM may also influence DNA DSB repair by modulating the processing of broken DNA ends. During V(D)J recombination, the general NHEJ enzymes, DNA Polymerase μ and λ, as well as the lymphoid-specific repair polymerase, terminal deoxynucleotidyl transferase, delete and add nucleotides at the coding ends, respectively [44], [45]. It remains to be determined if any of these enzymes is a substrate for the kinase activity of ATM or one of its downstream targets. Alternatively, the proposed function of ATM in stabilizing coding end breaks [16] may indirectly alter the junctional diversification process during end joining.

Taken together, the experiments presented here demonstrate that ATM is necessary for efficient TCRβ rearrangement in DN2/3 cells. When ATM is deficient, this defect in TCRβ rearrangement disrupts all subsequent stages of αβ T cell development as well as altering generation of the expressed TCRβ CDR3 repertoire.

## Materials and Methods

### Animals

ATMKO [22], TCRαKO [33], RAG1KO [46], and TCRβ TG [38] mice were maintained at Bioqual (Rockville, MD). C57BL/6, B6.CD45.1, and (C57BL/6×B6.CD45.1)F1 mice were obtained from Frederick Cancer Research Facility. Mice were used between 1.5–6 months of age.

### Ethics Statement

Animal experiments were approved by both Bioqual and National Cancer Institute Animal Care and Use Committees and assigned protocol numbers 09-3447-79 (Bioqual) and EIB-079 (National Cancer Institute).

### Antibodies

Steptavidin-PE-Cy5, streptavidin-FITC and Ab specific for CD4, CD8, TCRβ, CD44, CD25, B220, TCRγδ, Mac-1, NK1.1, and GR-1 were purchased from BD Biosciences (San Jose, CA). Rabbit anti-53BP1 Ab and Streptavidin-Alexa Fluor 594 were purchased from Molecular Probes (Eugene, OR) and Novus (St. Charles, MO), respectively. CD8-Alexa Fluor 647 and goat anti-rabbit IgG-Alexa Fluor 647 Ab were purchased from Invitrogen (Carlsbad CA).

### Cell Staining and Purification

Thymocytes were prepared, stained for flow cytometry, and analyzed as previously described [39]. For purification of DN cells, thymocytes were enriched for DN cells by CD4- and CD8-specific magnetic bead depletion (Milteny Biotec, Auburn CA). Cells were then stained with a lineage-specific Ab cocktail previously described (42) and anti-CD25 Ab, and sorted for lineage negative DN2/3 (CD25^+^) cells (>98% pure).

### Intracellular Staining

Thymocytes were first surface stained to identify DN cells and subsequently fixed and permeabilized (eBiosciences, San Diego, CA). Cells were then stained with either cleaved-Caspase-3 (Asp 175)) Ab (Cell Signaling Technology, Danvers, MA), to assess cell death, or DAPI, to assess cycling cells.

### Competitive Radiation Bone Marrow Chimeras

Radiation bone marrow chimeras were prepared as previously described [47]. Host (C57BL/6×B6.CD45.1)FI mice (CD45.2^+^×CD45.1^+^) were irradiated with 950 rad, and reconstituted with 6×10^6^ T cell-depleted bone marrow cells consisting of equal numbers of cells from ATMWT (CD45.1^+^) and either ATMWT or ATMKO (both CD45.2^+^) mice. Host cells (CD45.1^+^ CD45.2^+^), ATMWT donor (CD45.1^+^), or ATMKO and ATMWT donors (CD45.2^+^) were identified by staining for the indicated CD45 alleles. Chimeras were assayed 6–8 weeks after reconstitution.

### Quantitation of Vβ-DJβ Rearrangement by Real-time PCR

Genomic DNA was prepared from FACS-sorted lineage negative DN2/3 cells isolated from pools of 5–7 ATMWT or KO thymi. Specific Vβ-DJβ rearrangement combinations were amplified from 40–75 ng of genomic DNA using RT^2^ SYBR® Green qPCR Mastermix (Qiagen, Valencia, CA). Primer sequences: BV3F: CCTTCAAACTCACCTTGCAGC, BV5F: CCCAGCAGATTCTCAGTCCAAC, BV6F: TCTGCCCAGAAGAACGAGAT, BJ1.1R: ACTGTGAGTCTGGTTCCTTTACC, BJ1.4R: GACAGCTTGGTTCCATGACCG, BJ2.1R: GTGAGTCGTGTCCCTGGTCCGAAG, BJ2.7R: CTAAAACCGTGAGCCTGGTGC.

Reactions were run on a 7900 HT Fast Real Time PCR System (Applied Biosystems, Carlesbad, CA) and analyzed with SDS 2.3 software (Applied Biosystems). For each sample the Vβ-DJβ reactions were normalized to the invariant, non-rearranging region of the TCRα locus (Cα) using the following formula: 1.9^−CtV−Jβ^/1.9^−CtCα^. Amplification products were verified by dissociation curve analysis and gel electrophoresis.

### Immuno-FISH

Immuno-FISH was used to quantify DSB at TCRβ loci from FACS–sorted lineage negative DN2/3 cells prepared from pools of 5–7 ATMWT or KO thymi or from individual RAG1- deficient ATMWT and KO whole thymi (three of each genotype) [48]. Slides were stained with rabbit anti-53 BP1 Ab, washed, and stained with goat anti-rabbit IgG-Alexa Fluor 647 Ab. After chemical crosslinking and DNA denaturation a denatured TCRβ-specific FISH probe (RP23 284 D11 BAC (CHORI, Oakland, CA)) labeled with biotin-conjugated dUTP (Roche, Pleasanton, CA) was added. Slides were hybridized, washed, and stained with streptavidin-FITC. Imaging was done using a Zeiss LSM 510 Meta confocal microscope with a 63× objective and LSM imaging software. Zeiss AIM software was used for blinded analysis of 53 BP1 focus co-localization with TCRβ FISH signals.

### 3D-FISH

rted lineage negative DN2/3 cells prepared from pools of 5–7 ATMWT or KO thymi as previously described [32], [49]. After DNA denaturation, denatured TCRβ-specific BAC (CHORI) FISH probes were generated by labeling with either Spectrum Orange (Enzo, Farmingdale, NY)–labeled dUTP (Roche) (5′ TCRβ probe; RP23 284 D11) or Cy5 (GE Healthcare)–labeled dUTP (3′TCRβ probe; RP24 299 B14). Slides were hybridized overnight and imaged using a Zeiss LSM 510 Meta confocal microscope with a 63× objective and LSM imaging software. Single cells images were imported into the Imaris imaging program, where the center of homogeneous mass was calculated for each individual FISH signal. Using the X, Y, and Z coordinates obtained, distances between spots were calculated using the following formula for Euclidian distance in 3 dimensional space: 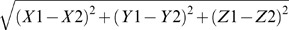 where X, Y, and Z denote the spatial coordinates within the nucleus for spot 1 and spot 2 [30].

### High Throughput Sequencing of TCRβ CDR3 Regions

FACS-sorted DP cells were prepared from ATMWTTCRαKO and ATMKOTCRαKO mice. Naïve CD4^+^ T cells (CD4^+^, CD44^−^) were FACS-sorted from ATMWT and ATMKO spleens. Genomic DNA was isolated from the sorted cells and deep sequencing of TCRβ rearrangements was performed by ImmunoSEQ™ sequencing (Adaptive Biotechnologies, Seattle WA). This multiplex PCR system amplifies all possible V-Jβ combinations and identified between 85,000 and 140,000 unique TCRβ V-D-J junctional sequences for each sample. For each sample the number of nucleotides added or deleted from each germline sequence was determined using ImmunoSEQ™ Analyzer software. Access to this sequencing data can be obtained by contacting SB or KSH.

### Statistical Analysis

Data significance for all experiments was tested using Student’s t-test using Excel software. For individual experiments, specific comparisons are indicated in figure legends. When indicated, mean±SEM is graphed and *p<0.05; **p<0.01; ***p<0.001.
